# “We are Forgotten”: Forced Migration, Sexual and Gender-Based Violence, and Coronavirus Disease-2019

**DOI:** 10.1177/10778012211030943

**Published:** 2021-09-17

**Authors:** Jenny Phillimore, Sandra Pertek, Selin Akyuz, Hoayda Darkal, Jeanine Hourani, Pip McKnight, Saime Ozcurumez, Sarah Taal

**Affiliations:** 1 Social Policy, 1724 University of Birmingham Edgbaston Campus, Edgbaston, Birmingham, UK; 2 52948 Department of Political Science and International Relations, TED University, Turkey; 3 Global Health, University of Melbourne, Melbourne, Victoria, Australia; 4 Refugee Women Connect, Manchester, UK; 5 Baobab Women’s Project, Birmingham, UK

**Keywords:** COVID-19, pandemic, structural violence, forced migrant women, SGBV

## Abstract

Adopting a structural violence approach, this article explores, with survivors and practitioners, how early coronavirus disease-2019 pandemic conditions affected forced migrant sexual and gender-based violence survivors’ lives. Introducing a new analytical framework combining violent abandonment, slow violence, and violent uncertainty, we show how interacting forms of structural violence exacerbated by pandemic conditions intensified existing inequalities. Abandonment of survivors by the state increased precarity, making everyday survival more difficult, and intensified prepandemic slow violence, while increased uncertainty heightened survivors’ psychological distress. Structural violence experienced during the pandemic can be conceptualized as part of the continuum of violence against forced migrants, which generates gendered harm.

## Introduction

The emergence of the coronavirus disease-2019 (COVID-19) pandemic has had an impact on populations across the globe, but not all populations are affected equally. Preexisting health and socioeconomic inequities shape people's vulnerability to the disease, exacerbating unequal societal structures as they determine inequitable health and socioeconomic outcomes across different members of society. For instance, in the UK and the US, higher rates of infection and mortality are evident for Black, Asian, and Minority Ethnic groups (Godin, 2020). Emergent literature (Bouillon-Minois et al., 2020; Bradbury-Jones & Isham, 2020; Roesch et al., 2020) highlights an association between lockdown and increased intimate partner violence (IPV) which places women at greater risk of harm. Concerns have been widely expressed that forced migrants, many of whom live in crowded, sometimes makeshift accommodation with poor access to food, sanitary items, and healthcare and are dependent on nongovernmental organizations (NGOs) or the informal labor market (Aultman, 2019), may be at particular risk, perhaps unable to access the materials and care they needed to stay safe from infection. Yet, experiences of forced migration are highly variable, dependent on age, gender, immigration status, and context (Menjívar & Perreira, 2019). Women survivors of sexual and gender-based violence (SGBV) are among the most vulnerable forced migrants frequently at risk of violence across the refugee journey (Ko & Perreira, 2010), including once in refuge or post-resettlement, with the very credibility of the abuse experienced questioned and shown to undermine claims for protection (Baillot et al., 2014). Women who have experienced multiple traumas may be at continued risk of violence and may receive insufficient protection from COVID-19-related social, economic, and health risks.

The vulnerabilities and insecurities of forced migrant women require a deeper analysis of the current crisis. Intersectionality theory highlights that violence against women is rooted in multiple systems of oppression and inequalities (Crenshaw, 1991). Migrant women often face disadvantages and discrimination on the basis of their gender, legal status, race, and other contextually marginalized social characteristics. An intersectional engagement alerts us to a critical understanding of migrant vulnerabilities and subordination, including different forms of precarity varying from immigration status, unpaid work, insecure employment, stereotypes, and prejudices about refugee women (Şenses et al., 2020). Gender-blind discourses of forced migration experiences, from displacement to resettlement and often gender-unresponsive provision of services, fail to capture or address multiple risks and threats to refugee women's safety and security in their socioecology at both interpersonal and structural levels. As a result, unacknowledged migration-related vulnerabilities extend patriarchal power imbalances, compounding a continuum of violence in migrant experiences (Canning, 2020). Prepandemic, scholars highlighted the need to adopt an intersectional approach to address barriers to services (Asgary & Segar, 2011), lack of assistance (Adams & Campbell, 2012), information gaps (Wachter et al., 2020), and challenges to SGBV-informed service provision and policies (Oliveira et al., 2018). COVID-19 conditions may produce different or intensified vulnerabilities for forced migrant women and especially the survivors of SGBV.

Adopting what Henderson (2020) describes as a structural violence approach, this article responds to the question of how did early pandemic conditions shape the lives of forced migrant survivors of SGBV? To answer this question, we introduce a new analytical framework bringing together three forms of violence: violent abandonment (Schindel, 2019), slow violence of the everyday (Mayblin et al., 2020), and violent uncertainty (Grace et al., 2018). We show how these interacting forms of structural violence are exacerbated by pandemic conditions but operate as part of a continuum of structural violence, which as Galtung (1969) contends, place survivors at risk of personal violence. Based on interviews with forced migrant SGBV survivors and service providers operating in five countries, we examine multiple accounts of the impact of the public health emergency, focusing on how the conditions affected women survivors’ everyday lives. We show that during the emergency, survivors were largely abandoned by states, with service providers struggling to reach those most in need as they closed services or shifted to remote provision. Abandonment increased precarity making everyday survival more difficult and exacerbating the slow violence experienced prepandemic, while increased uncertainty heightened psychological distress. The risk of physical harm intensified under pandemic conditions, which generated dependency on and placed them in close proximity to, perpetrators, while escape routes closed and psychological distress led some survivors to contemplate self-harm (UNFPA, 2020; UN Women, 2020).

We begin the article by outlining the background to the study stressing the importance of a focus on SGBV survivors and their experiences before presenting our analytical framework connecting three forms of structural violence. We then describe the situations in case study countries and set out the methods utilized before engaging with data from the interviews to demonstrate how pandemic conditions exacerbated structural violence for survivors, addressing the three forms of violence in turn, before showing how the accumulation of the violence increased survivors' risks of personal violence. We finally argue for the importance of understanding the multiple, cumulative, and interacting nature of different forms of structural violence, and the need for greater support for SGBV survivors in times of crisis to avoid exacerbating vulnerability.

## Background

Persistent inequalities have intensified the movement of people across borders in the past two decades. Yet, the category of forced migrant denotes a bureaucratic “label,” which has historically identified for public authorities, international organizations, and NGOs, a group for whom they should provide services (Zetter, 1991). Scholars debate whether states have an obligation to allow such individuals to cross their borders freely and to protect those who arrive by providing them equal access to services in the receiving country (Schmidtke & Ozcurumez, 2008; Wellman & Cole, 2011). The phenomenon of SGBV as well as its social construction and legal implications in the forced migration journey are embedded in this conceptual and empirical contestation. Addressing SGBV-related needs entrenched in social, economic, and political vulnerabilities, which both predate and occur across the forced migration journey, is challenging. Responsibility for and the funding of service provision are contested.

Forced displacement has reached an all-time high (UNHCR, 2020). Until relatively recently, Canada, the UK, and the US were major destinations for resettlement of those recognized as United Nations High Commissioner for Refugees Convention refugees. However, the advent of the Syrian conflict in 2011 generated flows of forced migrants, who are persons subject to migratory movement underpinned by an element of coercion (IOM, 2011), of a scale not seen since World War II. Countries adjacent to Syria received millions of forced migrants over a short period of time. At the same time, flows of individuals coming via Northern Africa into Southern Europe were increasingly problematized (Cusumano, 2019) as most liberal democracies became increasingly unwilling to accommodate large numbers of forced migrant arrivals (Holmes & Castañeda, 2016). In the post-2015 period, outsourcing forced displacement, already an important mechanism of immigration control, has been a major strategy for many countries of resettlement. This is particularly the case for European Union (EU) member states who operate within what Hall (2017) has described as a brutal migration milieu in which migrants are refuted and subject to punitive border regimes and normalized discriminatory sorting sustained by a historically rooted ethos of subordination. As such, EU countries have contracted with countries such as Turkey and Tunisia, which subsequently became simultaneously countries of transit, refuge and resettlement, wherein forced migrants live in a permanent state of exception. State responses to forced migration revealed several inadequacies in emergency preparedness and response in humanitarian crises, particularly falling short in protecting women and children from SGBV and which might be considered as part of a continuum of structural violence at a time when a rupture, rather than an intensification of, the brutal migration milieu is necessary to ensure protection.

SGBV includes rape and sexual assault, as well as physical, psychological or emotional violence; forced marriage; forced sex work; and denial of resources, opportunities, services and freedom of movement on the basis of socially ascribed gender roles and norms (Interagency Standing Committee, 2005). The prevalence of different forms of SGBV, including IPV, increases during humanitarian emergencies (WRC, 2016). The extraordinary levels of SGBV experienced by forced migrants during recent conflicts, throughout flight, in temporary camps, and in immigration detention centers have been highlighted (WRC, 2016). The term SGBV enables us to look at violence from a gendered perspective and to acknowledge that women and children are the main, although not the only, targets. Forced migrants are vulnerable during flight, with UN Women (2013) noting harassment of women and girls. Smugglers often force women and children into sexual relations as a form of payment (Amnesty International, 2013). So-called transactional sex has been documented, as women and children are expected to perform sexual acts in exchange for food or safety (Freedman, 2016). Refugee camps are not designed with personal safety in mind, so women and children are either confined to their tents or at risk of being coerced into sex work (Anani, 2013; Freedman, 2016). Krause (2015) uses Kelly’s (1988) concept of the continuum of sexual-based violence to make the case that SGBV against forced migrants represents a continuum of violence wherein different forms of SGBV are connected across scope, forms, and conditions of violence and throughout conflict, flight, and displacement. We argue that the continuum of violence includes the structural violence which takes place in countries of refuge or resettlement. Social, political, and economic factors in local resettlement contexts continue to shape women's vulnerability to both structural and physical violence and are intensified by pandemic conditions.

SGBV against women and children has been documented in countries of asylum and resettlement. Having sought sanctuary, forced migrants are frequently housed in makeshift, overcrowded accommodation. A lack of single-sex or secure facilities leaves forced migrants vulnerable, with cases of sexual abuse at the hands of other forced migrants, staff, guards, and volunteers reported (WRC, 2016). The risks that camps pose to women's safety and security have been well documented (Amowitz et al., 2002; Grabska, 2011; Khawaja, 2004; Krause, 2020). Grabska in her analysis on gender mainstreaming in refugee camps argues that hierarchical power relations that define the camps' settings and especially the homogenized view of refugee women as victims and men as perpetrators exacerbate “not only gender asymmetries but also put women at risk” (Grabska, 2011, p. 81). Turner, too, discusses homogenized gendered (and also binary) visions of refugees in humanitarian responses to the needs of Syrian refugees (Turner, 2019). The perceived “vulnerability of women” and “security risks posed by refugee men” obscure sustainable protection services for refugees of all genders (Turner, 2019, p. 611). Hence, other than “visible” risks in situations of encampment (lodging assignments, lack of security and privacy, lack of reporting channels), broader gendered power structures compound inequalities. Forced migrant women and their children report family violence throughout the process of resettlement and struggle to access services that do not account for the complexities of forced migrants’ SGBV experiences (Vaughan et al., 2015). The full extent of SGBV is unknown with incidence underreported (UN Women, 2013). The experience of SGBV is mentally and physically debilitating and can prevent individuals from rebuilding their lives, potentially further exacerbating the socioeconomic inequalities that characterize resettlement in countries of reception. Experiences of SGBV have tended to be viewed as personal, rather than socially and politically generated acts, and thus an individual problem rather than a state concern (Krause, 2015). Despite considerable evidence provided by NGOs, little political or policy attention has been paid to SGBV, its treatment, and the long-lasting consequences for forced migrants.

Humanitarian actors have highlighted the need to provide immediate assistance to survivors. However, the scale of recent emergencies has not been matched with the appropriate resources, capacity, political will, or governance models to enable the development of gender-sensitive services and facilities. Thus, those who have experienced SGBV lack the opportunity to seek protection or treatment and instead are frequently discriminated against because of punitive border regimes. Victims/survivors of SGBV are susceptible to severe psychological distress. Experiences in detention, authorities’ culture of disbelief, and the insistence on repeated retelling of SGBV experiences during asylum-seeking processes, can further traumatize individuals (Medical Foundation, 2004). The consequences of SGBV can be profound if treatment is not received or is inappropriate. Undisclosed SGBV (Pittaway & Bartolemei, 2001) and associated posttraumatic stress disorder (PTSD; Phillimore, 2011) may account for forced migrant women's poorer health outcomes compared with men's (Cheung & Phillimore, 2017). Given the long-term emotional–psychological (O'Doherty et al., 2015), socioeconomic (Keygnaert et al., 2015), physical (WHO, 2013), and sexual reproductive (O’Doherty et al., 2015) consequences of SGBV, it is important to consider strengthening the role of policy and practice in preventing or reducing incidents. This is especially the case when the conditions of the refugee emergency are exacerbated by the public health crisis of the COVID-19 pandemic. Given the acknowledged vulnerability of women forced migrants and the particular health issues faced by SGBV survivors, it is important to examine how they fare in what might be described as a double crisis.

### Structural Violence and Forced Migration

Galtung (1969) introduced the term “structural violence” to highlight the structures of inequality that comprise forms of violence, arguing that these can also lead to personal violence. For forced migrant women, such forms of violence can exist side by side as part of what has been described as a continuum of gender-based violence (Roupetz et al., 2020). Structural violence refers to harms that are preventable and where no one actor (person) commits the violence, but rather the harms emerge from inequality built into structures. This article focuses on three types of structural violence, which have been discussed in relation to forced migration: violent abandonment, slow violence, and violent uncertainty. The article examines how in the context of the COVID-19 pandemic (hereon, the pandemic) this violence accumulates to shape the lives of forced migrant survivors of SGBV as part of a continuum of structural violence, where existing vulnerabilities are exacerbated and, in some cases, increase the risks of personal violence. Writing about unaccompanied young people, Menjivar and Perreira (2019) refer to cumulative violence and trauma that occur across the refugee journey, highlighting an accumulation of inequalities and disadvantages that occur over time and place with gender being a key dimension in inequality. We point to the cumulative nature of violence over time and place but also multitudinous forms of violence that forced migrant SGBV survivors face, due to intersectional inequalities, which operate as part of the continuum of violence that begins with displacement and continues while women are out of place.

Violent abandonment refers to inaction in the face of forced migrant suffering and how it is used as a mechanism of control. Herein we refer to the abandonment of SGBV forced migrant survivors by states. Such an abandonment takes place not only over time and space but also in relation to specific situations. Schindel (2019) outlines how state violence was previously confined within territorial borders, with the potential of being enacted in every border check, but has become “*spatially expanded and continuous: a violence discharged slowly upon persons forced to be on the move for longer durations*” (p. 9). Migrants are forced on ever longer and more dangerous trips that are known to be particularly risky for women, involving heightened risks of SGBV. They are forced to move “illegally” through difficult topography with the goal of inducing (unending) movement invoking new forms of control that are not fixed to a space. Abandoning migrants to nature, rather than confronting them at the border, shifts violence from the visible to the invisible, so it is at once bureaucratized because it is state sanctioned, indirectly pushing migrants into spaces of “civil unprotection.” Risks are deliberately spiraled by states who force migrants to take increasingly dangerous routes in a strategy intended to prevent them from arriving. Thus, the refugee journey in itself and the failure to offer protection or services until forced migrants cross a designated border constitute abandonment. Yet, as Davies et al. (2017) show in relation to the Calais camp, “the Jungle” in Northern France, even after they have arrived at an imagined space of refuge, active inaction through the intentional withholding of care continues the abandonment. They argue that abandoning migrants to the Calais camp in conditions that are harmful, both physically and mentally, and depriving them of food, medicine, and sanitation, leaves individuals destined to suffer a socially sanctioned dehumanization where states’ failure to act normalizes suffering. They argue migrants are “not actively killed—as would befit a ‘bare life’ reading—but destined to suffer the harm and indignity of long-term cruel conditions” (p. 1280).

To some extent, the abandonment of migrants by the state that Schindel (2019) describes is a form of slow violence in that it unfolds over time, implying a temporal dimension to violence. Slow violence can be conceived of as everyday violence experienced over time and designed to subjugate. The term originates from Nixon’s (2011) work on environment where violence is invisible, dispersed across time and space, attritional, and not generally conceived of as violence: “a violence that is neither spectacular nor instantaneous, but rather incremental and accretive, its calamitous repercussions playing out across a range of temporal scales” (p. 2). Mayblin’s (2019) analysis of the everyday lives of asylum seekers in the UK shows how structures in the form of social policies can play out as slow violence. She refers to the illegally low level of support provided to asylum applicants, which is not unique to the UK, and how a combination of the stress of everyday survival on minimal resources and the endless waiting for a decision on their asylum claim, a situation we consider below as violent uncertainty, was harmful. Mayblin (2019) describes how her subjects engaged in a constant endeavor of survival with time spent trying to find the cheapest foods to eat, the need to share resources with others to reduce costs by buying in bulk, and the importance of reciprocity in times of food shortages. She describes dilemmas faced choosing between personal hygiene, clothing, and food, and the immobilizing effect of the asylum system in denying individuals funds to use public transport and containing people in local areas. She shows how relationships with other asylum seekers are lifesaving, both in terms of support offered through shared resources and the shared experience of hunger, shame, and stigma. Ultimately, these small everyday harms are shown to be physically and psychologically injurious.

Waiting is a condition of both seeking asylum and resettlement (Conlon, 2011), which Auyero (2011), building on Bourdieu (2000), describes as an act of subordination. While the relationship between waiting and forced migration has long been established, Grace et al. (2018) have coined the term violence of uncertainty to capture the relationship between policies of uncertainty and the systematic and harmful insecurity that they generate. They describe multiple fears: of deportation, separation from children, and discrimination, and increased reluctance to use health care services encountered by clinicians noting increased levels of PTSD in response to uncertainty. Qualitative evidence from across Europe and Australia suggests a strong connection between forced migration, uncertainty, and health. Inability to influence the asylum process and lack of control over one's life combined with concerns about the general population's views of asylum seekers left individuals lacking self-esteem and feeling humiliated (Cleveland et al., 2018; Lauritzen & Sivertsen, 2012). Fear of removal (Sourander, 2003) and being separated from family (Reesp, 2003), exacerbated by the absence of distractions (Lauritzen & Sivertsen, 2012), have also been reported. Uncertainty and fear are said to have a greater impact on asylum seekers than premigration trauma (Lauritzen & Sivertsen, 2012) and result in wide-ranging mental health problems such as depression, anxiety, psychosocial distress, and suicide ideation (Schoretsanitis et al., 2018). Some scholars point to adversarial asylum systems, poor decision making, and the questioning of credibility of rape and other claims as being problematic for individuals’ health, as individuals are kept in a profound state of uncertainty in which their lived experience is denied (Baillot et al., 2014; Beneduce, 2015; Rotter, 2016). Certainly, Auyero (2011) finds obfuscation of processes so that the very basis on which decisions are made is unclear and appears arbitrary, which further subjugates those who wait. Flawed decision making leaves people in limbo, in an open-ended state of waiting described as punitive (Rotter, 2016). The violence of uncertainty is both slow, in that it is experienced over time, often many years with no clear end point, but also involves uncertain endpoints, including abandonment when claims are denied and periods of destitution and homelessness ensue (Chase, 2020). This intertwining structural violence are now well understood in relation to forced migration and there is increasing evidence regarding their gendered nature. We now move on to consider the methods engaged to explore how such violence is both continued and exacerbated under pandemic conditions.

## Methods and Ethics

The data presented in this article were collected as part of a study that aimed to identify the effects of COVID-19 on forced migrant SGBV survivors and the organizations that support them. The study built upon a multicountry study of SGBV and forced migration known as the SEREDA project. We invoke the term forced migrants to cover all individuals who have been involuntarily displaced from their homes, focusing on the experiences of displacement rather than on specific and frequently contested statuses, noting that some statuses, in intersection with other factors such as age, make some migrants more vulnerable than others. We sought to urgently collect data about the effect of the crisis to highlight the impact of the pandemic on survivors to policymakers (see Pertek et al., 2020) returning, where safe, to all individuals previously interviewed for the SEREDA project enabling speedy access to respondents. Such individuals were originally selected for inclusion in the project on the grounds that they were adult forced migrant SGBV survivors and identified through contact with gatekeeper NGOs, personal networks, word of mouth, and snowballing. In a two-week period between April 14 and 28, 2020, we interviewed 52 survivors and 45 service providers in five countries (Tables 1 and 2): the UK, Turkey, Tunisia, Sweden, and Australia. The rationale for the inclusion of these countries included that they consisted of a number of destination and transit countries with different forced migration resettlement regimes (see Appendix 1). At the time of the interviews, responses to the pandemic differed, with all but Sweden operating some form of lockdown and social distancing encouraged throughout.

**Table 1.** Demographics of Survivors Participating by Country.

		Sweden	Tunisia	Turkey	UK	Subtotal
Gender	Female	6	11	11	20	48
	Male	3	1			4
Total		9	12	11	20	52
Age	20s	1	4	3	3	11
	30s	4	7	6	11	28
	40s	4	1	1	4	10
	50s			1	2	3
Total		9	12	11	20	52
Country/region of origin	Albania				2	2
	Cameroon		1		1	2
	Congo		2			2
	Eritrea		1		6	7
	Gambia				1	1
	Ghana		1		1	2
	Guinea		1		2	3
	Iraq	2				2
	Lebanon	1				1
	Malawi				1	1
	Namibia				2	2
	Nigeria		4		4	8
	Sierra Leone		1			1
	Sudan		1			1
	Syria	5		11		16
	Turkey	1				1
Total		9	12	11	20	52

**Table 2.** Category of Service Providers Participating by Country.

	Australia	International	Sweden	Tunisia	Turkey	UK	Subtotal
Local nongovernmental organization (NGO)	5			3		3	11
Regional NGO						4	4
National NGO	2		1	3	5	4	15
International organization		2		3	4		9
Public institution			3		1		4
Faith-based						1	1
Private						1	1
Total	7	2	4	9	10	13	45

Survivor respondents ranged in age, ethnonational background, and legal status. All but four were women. Some 30 originated in sub-Saharan Africa (two from Cameroon, two from Congo, seven from Eritrea, one from the Gambia, two from Ghana, three from Guinea, one from Malawi, eight from Nigeria, two from Namibia, and one each from Sierra Leone and Sudan). The remaining 22 originated in the Middle East and North Africa region (16 from Syria, two from Albania, two from Iraq, and one each from Lebanon and Turkey). We asked survivors one question, “*How has the COVID-19 emergency affected your life?*” and probed across different areas, including their socioeconomic situation, food security, work, social connections, access to welfare and health care, and their wellbeing. Service providers ranged from international, national, and local NGOs, public institutions, and private service providers working with forced migrants. Respondents included NGO directors, migration and protection program managers, SGBV personnel, psychologists, doctors, and social workers. We asked service providers about the impact of the crisis on survivors and on the support services providers offered.

Emergency ethics approval was gained from the University of Birmingham Ethics Committee, as an extension of the approval for the SEREDA Project. We contacted those survivors we knew were living alone by telephone message or WhatsApp, taking care to reach out only to those whom we knew, from previous interactions, were safe to pick up messages. Interactions commenced with questions aiming to ascertain safety and a discussion of the project aims and objectives before requesting informed verbal consent prior to the start of phone interviews. When using email and WhatsApp, respondents were sent information about the project and asked to consent before responding. Interviews were undertaken by the research team in Arabic and English across countries, while interpreters with experience working with forced migrant survivors assisted with data collection in Turkey and Tunisia. In Australia, the survivors we had already interviewed were largely still partnered, potentially presenting safety risks should they be recontacted for an interview under lockdown conditions. Therefore, no survivor interviews were undertaken in Australia.

Data were collected in various forms: email and WhatsApp responses, verbatim recordings of interviews, and some interview summaries, which included notes of key issues emerging from conversations. All data were anonymized and stored in an encrypted data storage facility. To undertake the data analysis, we used a systematic thematic approach, identifying key themes and using these to create an analysis grid to condense, process, and compare data (Guest et al., 2012). We engage with both the stakeholder and women survivor data below.

## Findings

### Abandonment in the Emergency

In each of the countries, different measures were introduced, including increased social assistance, job support schemes, and support to access online services. These were invariably directed at citizens. Many SGBV survivors in all countries were heavily dependent before the pandemic on conditional support via either asylum support schemes, welfare provision, or NGO or humanitarian assistance. The nature of support varied enormously from small amounts of cash, vouchers, food, shelter, and psychosocial support. Service providers recognized that moving to remote service delivery meant they were unable to reach all those in need. Immigration status emerged as a prepandemic factor, which hugely impacted access to resources as the pandemic evolved. Destitute forced migrants with irregular immigration status were seen as, and reported, facing particular difficulties as the charities that supported them closed their doors. The suspension of psychosocial support activities removed the only lifeline for some survivors, which significantly undermined their wellbeing.

My level of anxiety and uncertainty has increased because of the self-isolation. I don't do what I usually do, I can't attend my support groups, I can't meet my friends as I usually do. So, I am confined in my room which makes it really, re­­­ally, really difficult for me. (Valentine, Cameroon, asylum seeker, 40s, UK)

Many empowerment programs on which survivors relied to aid recovery also ceased operation. In the UK, NGO coalitions advocated for cessation of medical charges for forced migrants with no recourse to public funds (NRPF)^1^ with exemptions granted for COVID-19 but no other conditions. Respondents with NRPF status, who were already subject to high levels of conditionality in access to welfare, were unable to access public emergency accommodation or food vouchers and were pushed toward destitution. Forced migrant workers working in agriculture (Turkey), with zero-hour contracts (UK), and with no employment contracts (Tunisia) were unable to access any state benefits and were excluded from the safety nets implemented for the general population, leaving them in highly precarious situations without any support. Closure of government facilities caused delays with issuance of new and renewed visas and residence permits. In Turkey, the closure of such facilities meant documents became invalid, work permits could not be renewed, and forced migrants became “illegal.”

As of the latest updates, the Work Permits of Foreigners has extended, renewal of Residence Permits has been delayed and scheduled, but the issuing of Temporary Protection has been suspended til June (Executive Director, Local NGO, Turkey)

Many participants living in the UK, Tunisia, and Turkey described inadequate living conditions that intensified during lockdown with no measures introduced to help them live safely. Forced migrants living in shelters, shared accommodation, and overcrowded housing with shared kitchens and toilets were unable to self-isolate. Living in overcrowded facilities generated health risks and anxiety about contracting the virus from co-residents, who went in and out of shelters without protection.

We are almost 120 people … we have 20 rooms, so in each we have 10 to 12 people … they are not family … we have bathrooms in our rooms … there is no isolation, when you open to door you must jump over everybody … people go outside and come back to the same shelter … they just made it worse for us … every 4 pm they lock the gates, but normal time is 6 pm (national lockdown guidelines) … so we are not safe, we are here up to 120 people and everyone leaves in the morning, so where is the isolation? (Adam, 20s, Nigeria, asylum seeker, Tunisia).

Some migrants were threatened with eviction in Turkey and Tunisia after losing their jobs. Forced migrants living in shared accommodation in the UK lacked access to working washing machines, which were important for hygiene purposes. Also, in the UK, poor quality asylum housing was not repaired, with respondents believing they had been abandoned during the crisis.

Undocumented forced migrants were anxious about seeking medical help and fearful of unaffordable charges or being reported to immigration authorities and detained. Respondents found it difficult to access adequate information on COVID-19 from government websites, relying instead on multilingual information offered by NGOs. Some participants expressed concerns that national policies on COVID-19 were inequitable. Several asylum seekers in Sweden felt abandoned by the state, feeling that the policies adopted there did not take account of their vulnerability:

We don't have the same degree of immunity as the Swedes. The difficulties we experienced and the lifestyle we had in our countries are so different than the ones of the Swedes. The government knows that, yet still decided to follow different policy than the rest of the world. (Amel, 30s, Syria, refugee, Sweden)

Asylum seekers in the UK worried they would not be a priority for access to a ventilator if needed. Some respondents worried that mortality rates in the forced migrant population would not be recorded and thus they were invisible to authorities and did not matter. These beliefs reflect the ways in which border regimes subordinate forced migrants, resulting in symbolic violence in which migrants are made to feel they are of lower value than the citizenry (Gamlin, 2013).

In the UK and Tunisia, NGOs played an important role in mobilizing governments to support forced migrants during the pandemic and to include rather than abandon them. In Tunisia, an international NGO advocated for the Ministry of Human Rights to consider the situation of migrants. As a result, the issue of migration received the Government's attention and a three-month temporary regular status was granted. In the UK, the Government resisted these calls. NGOs also advocated for the provision of multilingual COVID-19 information, which was eventually provided in a small number of languages.

### Slow Violence of the Everyday—From Bare Lives to Violence of Hunger and Isolation

As we note above, many forced migrants lost sources of income and informal support, with no state welfare provision for those with irregular or international protection, leading to a worsening of already substandard living conditions, including hunger and dehumanization. Work previously available in the informal economy--for example, in retail, cleaning, and care sectors--disappeared. Women with children reported the stress of these socioeconomic burdens alongside increased care and domestic work as schools closed. They had to choose between food, hygiene items, and paying for mobile phone data to enable their children to participate in online schooling. Some did not have access to the hardware or software needed to facilitate children's education, and children living in crowded asylum housing had no space to study. In Tunisia, women were unable to afford milk and diapers for children, some born of sexual violence, and no support was provided for children below the age of eight months.

We got tired of talking all the time, nothing changes! IOM does not make any effort to help us as we are accommodated, for them it is enough, we are fine. Nothing works here; we have babies but there is nothing to eat, no milk or baby diaper. IOM gives us a 30 dinar voucher every week. What can that do to a person for a week? Is there any person who can have food that costs only 4 dinar per day? Since the confinement, we have received nothing. (Melanie, 30s, DRC, asylum seeker, Tunisia)

Respondents reported increased food prices and the need to switch to cheaper products or choose between essential goods such as food or hygiene products, with some women experiencing food shortages. In Turkey, stakeholder respondents advised that the most disadvantaged were forced migrants under International Protection who received no support. Forced migrants receiving vouchers were no longer able to convert them into cash as they used to with friends and which had enabled them to access a wide range of cheaper shops and top-up their phones (UK and Tunisia). Respondents without a bank account were excluded from online bill payments or using public transport, which shifted to accepting only bank cards (UK), compounding their immobility and preventing access to cheaper resources. Without savings, individuals were unable to stockpile food items and feared going hungry. Survivors in the UK frequently relied on food banks and charity support, which were reduced under pandemic conditions. Everyday life became more of a struggle with the survival strategies that individuals previously used to get through their normal lives undermined by pandemic restrictions and the failure of states to extend support to forced migrants and SGBV survivors.

In *section 4* I know you’re not going to get food from there, but they give me the voucher to get food, no money. The way it affects me if I don't have cash on me, you’re not allowed cash money, that way it affected me, if a commitment is important how I can get money to get bus. (Afafa, 20s, Ghana, asylum seeker, UK)

Many forced migrants living in shelters had limited access to television, internet, and phones. Digital poverty excluded respondents from participating in online support groups and some spent long days alone, sleeping, thinking about past experiences, and worrying about the future. Others were excluded from learning opportunities that had previously provided much-needed distraction from the respondents’ situation and also, as Auyero (2011) shows, as an important mechanism for dealing with uncertainty. Forced migrant survivors were deeply affected by social isolation, which exacerbated existing feelings of loneliness, sadness, and anxiety about income, health, and life. Forced migration uproots people from their homes and social connections with many having no family to rely on. As Mayblin (2019) shows, friendships are frequently experienced as lifesaving and some respondents had been developing social networks, which enabled the gradual rebuilding of hope, dignity, and confidence. Enforced isolation undermined their ability to maintain these nascent social networks, especially when their financial situation limited access to telephones or the internet. Extra solitary time compounded marginalization. The closure of places of worship deprived survivors of a key source of support. Reminders of the past affected their mental health and disrupted coping strategies. Anxiety and stress associated with the pandemic exacerbated existing concerns:

You know when you already have a problem and another comes on top it makes the problem bigger like when it's just one problem, but if you already have all those underlying things carrying this and that trauma, layers and layers of different things. (Nafi, 50, Malawi, asylum seeker, UK)

Many survivors compared the pandemic restrictions with those they experienced during earlier periods of abuse and exploitation such as enforced isolation, limited freedom, and no access to support. For those who experienced conflict and torture, lockdown activated painful memories of the self-isolation they deployed to protect themselves from violence. Some said they were forced to relive their traumas because of a lack of the distractions they usually employed to pass the time, such as going outside, meeting friends, and attending support groups.

### Extending the Uncertainty

The suspension of resettlement programs and asylum claims generated “the end of hope,” and what was hardly bearable temporarily became unbearable with the thought that processes had ceased altogether. In the UK, those awaiting asylum decisions had to wait even longer. Asylum processes shifted online or were processed by post, but service providers explained how in the pandemic it became more difficult for solicitors and risky for survivors, during intensified social isolation, to work with the traumatizing material needed to evidence their claims.

Sometimes I get low, cry, cry, cry, asking how long will this take, feel like I’m in a prison. You don't know what is going to happen, waiting for asylum decision and nothing is coming … Started asylum journey 2018 up until now 2020, no decision. Sometimes worried the traffickers will get my number, make more threats. Let me find my way to go on with my life, I want my life, so tired. (Maria, 40s, Nigeria, asylum seeker, UK)

Survivors in Tunisia, who were still in transit, considered the crisis as the worst thing that could happen to them after all the efforts they put into fleeing their countries as they became trapped by the lockdown without support. In Turkey, the disease was described by a survivor “as a bigger stressor than experiences of war and family abuse.”

Delays in processing survivors’ claims could be a source of relief, but generally delays added to their sense of despair. Halting bureaucratic asylum-seeking and resettlement processes meant putting lives on hold even longer and being overwhelmed with a sense of uncertainty, which hindered recovery from abuse and exacerbated the feelings of powerlessness Auyero (2011) shows are generated by pervasive uncertainty. In Tunisia, respondents were frustrated because the resettlement program was suspended and travel restrictions imposed. Survivors were stuck in exploitative situations, unable to flee amid pandemic restrictions. Delays in legal procedures and registrations with public authorities slowed down survivors’ access to assistance dependent on status (UK and Turkey).

When we started saving some money for our future plan, the Corona came to destroy everything. Since the outbreak of Corona, everything became so hard for us; as if we were frozen inside a refrigerator. Corona Virus put us in a big trouble. We can no more think or work for our future. We feel lost; especially, we, as foreigners who are stuck here. No one asks about us! (Senait, 30s, Eritrea, asylum seeker, Tunisia)

### From Structural to Sexual and Gender-Based Violence

The pandemic conditions exacerbated existing stressors particularly for marginalized individuals with no support. Many were already exposed to physical violence, which ran alongside everyday structural violence associated with uncertainty, abandonment, and slow violence in the continuum of violence that for some commenced preconflict. Nevertheless, the advent of pandemic conditions and particularly the intensified abandonment by the state increased the risk of physical violence. Service providers raised concerns that survivors locked in with perpetrators would lose the social and psychological resilience gained through the therapeutic work that they had provided prior to lockdown.

Abandonment, being outside of support programs, and losing work generated intensified precarity and dependency on perpetrators that increased vulnerability to physical violence. Service providers expressed concerns that some forced migrant women with NRPF status (UK) or temporary visa holders (Australia) were trapped between remaining in increasingly abusive or exploitative situations with violent partners or homelessness as they did not qualify for public housing or did not have access to existing services. There were reports of interpersonal violence increasing in these groups and a further rise in reporting was expected postlockdown (Australia, UK, and Turkey).

COVID's isolation challenges across mainstream society and it impacts on victims/survivors living with violent and controlling perpetrators are amplified for those that have cultural and linguistic barriers to help seeking, trauma backgrounds, visa insecurities, history of displacement and poverty. … (Project Coordinator, Local NGO, Australia)

Service providers noted an increase in reports of domestic violence associated with lockdown, suggesting that self-isolation at home was not safe for all. Yet, escape routes were restricted. In Turkey and Tunisia, shelters did not accept newcomers. In one Turkish municipality, the representative of the Women's Unit said that SGBV reporting increased threefold. In Turkey, economic hardship and lost jobs and income manifested in increased domestic violence also endangering children's safety. In Australia, while calls to a national helpline dropped 5%, there was a 20% increase in using an online chat service, indicating that women needed help but were unable to call from home. Calls to a men's helpline by perpetrators also increased notably. Survivors were said to be less likely to report violence and/or seek support, as perpetrators were more likely to be at home rather than at work. Reporting and help seeking during lockdown were also thought to be affected because survivors thought their suffering seemed trivial when compared with the threat of COVID-19 to their own and their children's overall survival. Service providers reported a decrease in the number of safe houses made available for women escaping violence. In the UK, survivors with NRPF status were ineligible for emergency accommodation, leaving them the option of remaining in an abusive relationship, or homelessness.

We have been concerned about the ongoing lack of emergency measures to support people with No Recourse to Public Funds during this emergency. This is leaving people with No Recourse to Public Funds trapped between remaining in abusive and exploitative situations or facing homelessness and destitution where they may be both at increased risk from their abusers after leaving and will be unable to follow government advice to keep themselves and others safe by “staying home” during this emergency as they have no access to housing and financial support. (Senior Policy and Advocacy Officer, International NGO, UK)

Service providers from different countries outlined how pandemic conditions increased the risks of forced migrants experiencing violence. In Sweden, for instance, one respondent believed that secondary school closures would increase the risk of honor-based violence, while in the UK, the risk of exposure to honor-based violence was said to deter domestic violence survivors from accessing support. Amendments to criminal law concerning the probationary release of SGBV perpetrators in Turkey increased risks of renewed perpetration and elevated survivors’ anxiety. On the other hand, in Tunisia, the issue of sexual abuse and exploitation in “fake” marriages was reported as a growing concern. Adolescent girls agreed to pretend they had marital relations with men they met in Libya, having been told that couples would access better protection and an expedited resettlement procedure. During the emergency, girls became trapped in these relationships and faced additional violence and criminalization when forced by their “partners” into prostitution, breaking pandemic restrictions.

When arrived to Tunisia, they are put together in one room and in this case, they found themselves raped and abused without their consent and they cannot report that because they have already lied concerning the reality of their relationships. Many of them give birth to children from their “partners.” (Coordinator, Local NGO, Tunisia)

Pandemic conditions and the lack of distractions provided by work, education, or NGO support services also intensified the risk of physical self-harm. Many survivors disclosed flashbacks, self-harm, anxiety, frustration, sleeping difficulties, and eating disorders exacerbated by social distancing measures. For some, the pandemic conditions generated suicidal thoughts:

Because of past violence, sometimes it makes me get those suicidal thoughts, I don't know how to explain it. You know when you feel like you are stuck. Start getting flashbacks, frustrated, start thinking too much. … Where you are out there, you don't think too much when you stay one place you start feeling, those flashbacks started coming back. (Assiatou, 40s, Guinea, asylum seeker, UK)

## Discussion

The continuum of violence has been used in studies of sexual violence, interpersonal violence, and conflict and peace as a metaphor to consider the wide range of gender-based violence (Kostovicova et al., 2020). It offers scope to enable us to think about continuity and change of circumstances shaping continued SGBV exposure. Our analytical framework has enabled us to highlight how, under pandemic conditions, forced migrant women, already vulnerable to SGBV, experienced an accumulation and intensification of structural violence that not only exacerbated their existing vulnerabilities and insecurities but also increased vulnerability to personal violence in a continuum of violence that spanned from premigration, conflict and resettlement, individual and state levels, and public and private realms. The pandemic added a further complexity to the intersecting inequalities and disadvantages that Menjivar and Perreira (2019) note happen to forced migrants over time and place. Forced migrant survivors were abandoned by the state with the loss of support services, income-generation opportunities, and access to general support systems, with no actions taken to address the risky nature of their accommodation or to provide for the need for greater levels of sanitation, placing them at risk of infection and unable to stay safe. Not only were many abandoned economically, but they also felt abandoned psychologically and feared inadequate or denial of care if they became infected. The lack of effort made to share information about COVID-19 or to ensure forced migrants’ physical safety suggests their fears may have some foundation. Abandonment exacerbated the slow violence of the everyday which Mayblin (2019) shows is already the lived reality for many forced migrants.

Respondents outlined increased pressures on everyday survival, choosing between food, mobile phone data, and hygiene, with no provision of these by authorities. The disadvantages already experienced by forced migrant children were also intensified as they struggled to access online education and families experienced food shortages. Pandemic conditions interrupted survival strategies. Travelling to access cheap food, sharing, and voucher-swapping were not possible, meaning that survivors had access to fewer resources even before worrying about additional data and hygiene needs. Isolation was highly problematic given the need for reciprocity in survival strategies as well as the importance of networks for psychological survival (Mayblin, 2019). Thus, the pain of everyday slow violence was exacerbated given that it could not be shared and, as Lauritzen and Sivertsen (2012) demonstrate, without the distractions that are so important to be able to get by on an everyday basis and to help cope with the disempowerment of waiting (Auyero, 2011). Survivors experienced the intertwined violence of uncertainty and the everyday as time under pandemic conditions took on a different temporality and survivors had no idea how long they would be living alone or in risky conditions, with so little. Rotter (2016) notes the punitive nature of open-ended waiting associated with asylum processes, but before the pandemic survivors at least knew that processes were active. The suspension of these processes took away all hope.

The women in our study had faced continuous violence over long durations, having been abandoned by states over time and across lengthy journeys in spaces of civil unprotection (Schindel, 2019) wherein no one took responsibility for them in a continuum of violence that was structural and enabled SGBV. The active inaction they faced through the withholding of care during the pandemic via failure to ensure they had access to food, medication, data, and hygienic conditions intensified already normalized suffering (Davies et al., 2017). Thus, for survivors, the violence of the past was exacerbated in the present and took on multiple forms as they were abandoned and the abandonment intensified everyday struggles, risks, and feelings of uncertainty in the context of forced migration (Ozcurumez et al., 2019). Galtung (1969) talks of the inevitable connection between preventable structural violence, which if allowed to come about results in personal violence, while Schindel (2019) stresses the importance of looking at relations between slow and fast violence. In this study, we demonstrate empirically a direct connection between structural and interpersonal violence as they intersect and form part of a continuum of violence. First, the experience of intertwined forms of structural violence heightened existing trauma causing psychological pain and increasing the risk of self-harm. Second, failure to ensure survivors had access to food and safe housing caused physical and psychological suffering through hunger and fear. Third, the abandonment of survivors to perpetrators and loss of escape mechanisms gave women no choice but to endure interpersonal violence and exploitation.

The pandemic also exposed the nature of discrimination forced migrants face at the intersections of multiple inequalities. As we show, forced migrants’ vulnerability to violence was exacerbated by an interplay of factors related to immigration status, gender, and age. Inparticular, the interaction between their irregular immigration status and gender detrimentally affected their experiences. For women with NRPF, on temporary visas, or with international protection, lack of access to state benefits coupled with the increased burden of domestic and care work and decline of informal employment during the pandemic compounded their vulnerability.

Pandemic situations are complex and there may always be groups whose existing vulnerabilities, compounded by deficiencies in emergency preparedness and response, may cause them to suffer more than others. However, one major point of caution is to prevent suffering from happening intentionally or as part of emergency response plans. Some categories of forced migrants in all but Sweden were intentionally excluded from wider support mechanisms as part of the brutal migration milieu, which uses border regimes to discriminate against migrants (Hall, 2017). Further, NGOs were extremely vocal in highlighting the need to protect everyone in pandemic measures with their demands sometimes accepted, but mostly denied. Thus, we conclude that the structural violence which intensified for many forced migrants was intentionally constructed, as Schindel’s (2019) work on border regimes shows, with the explicit purpose of further disenfranchising individuals as part of ongoing strategies to dehumanize and control. Further, given the already disadvantaged position of SGBV survivors, as women and forced migrants of different backgrounds, the structural violence resulted in what Canning (2020) describes as “gendered harm.” Such strategies clearly serve a political purpose, but systematically denying basic human needs constitutes what Ho (2007) has described as a structural violation of human rights and, as such, demands attention at the global level.

## Conclusion

Our structural violence approach introducing an analytical framework connecting different forms of structural violence offered a novel and valuable way of exploring in detail the nature of structural violence experienced by forced migrants, but also explicated the relations between structural and interpersonal violence that are part of a continuum of violence. We argue that this framework has wider utility beyond pandemic conditions. We show how pandemic conditions have added an additional layer of disadvantage for forced migrant women survivors of SGBV, which results from civic inaction resulting in gendered harm. We also show that forced migrant experiences were shaped by multiple intersecting inequalities; those with irregular immigration status, without access to public funds, and with caring responsibilities fared worse. Our analysis was based on interviews that took place in two weeks in April at the early stages of the pandemic. Since that time, the situation has evolved constantly and it is likely that the experiences of forced migrants have changed. Further attention is needed from an intersectional perspective to examine in greater detail differences in experience by gender, ethnonational identity, and immigration status, and between different country and policy contexts as the pandemic evolves. Attention too is needed to consider the ways in which the continuum of structural violence can be disrupted. Lilja and Baaz (2015) use Vergès’ (1981) concept of rupture to argue that interventions are required to break with gender norms understood as the natural order and resulting in acceptance of SGBV. The unequal treatment of migrants and failure to take responsibility for the care and safety of SGBV survivors have become so normalized in the brutal migration milieu that dominates responses to imagined migration crises that a rupture in the milieu is needed. An alternative discourse is necessary through which people are treated as humans in need of care, whether or not in the midst of a global pandemic.

## Notes

1. In the UK, forced migrants with NRPF status are unable to access social welfare and not permitted to work. They rely on either support from other migrants or from charity. Some access work in the Black economy in highly precarious jobs where they tend to be exploited.

## APPENDIX

**Appendix 1.** Characteristics of the Comparison Countries.

Country	Asylum/refugee population	Reception and support	SGBV and asylum/refugees
Sweden	163,000 refugees applied for asylum in 2015, the majority from Syria, Afghanistan, and Iraq, including 70,000 children, half of whom were unaccompanied.	Initially offered automatic permanent residency, but now most are offered temporary residence. Access to Sweden's generous health and welfare systems and some specialist psychological support.	Centrality of gender equality to Swedish culture means SGBV perpetrated by refugees is highly contentious. Claims made that some refugee cultures are inherently sexist. Discussion of rape and migrants politically problematic.
UK	Received around 25,000 asylum seekers arriving spontaneously per annum of whom ∼33% receive refugee status. Commitment to take 20,000 of most vulnerable Syrian refugees directly from camps and accept 3,000 unaccompanied children from Europe.	Refugees have the same entitlements as the general population and are allocated individual advice and guidance for 12 months after arrival. Asylum seekers can access health and limited benefits while their claim is being processed.	Dispersal programmes send individuals to areas unfamiliar with refugees and lacking specialist services. High levels of antirefugee sentiment are evident and racist harassment is widely experienced.
Australia	Humanitarian programme settles around 14,000 refugees per annum including Women at Risk visas. Asylum seekers arriving by boat are detained in offshore camps. In 2015, the government agreed to settle a further 12,000 people direct from the Levant prioritizing women, children, and families.	Arrivals through the Humanitarian Programme are entitled to access health and welfare services, with social and educational interventions. Asylum seekers have limited entitlements. Those in offshore facilities lack employment opportunities or access to specialized services, including support for SGBV.	Refugees are resettled in diverse locations across the country where services have limited capacity to address complex needs. State governments have expressed readiness to provide support to the additional cohort of refugees arriving from the Levant, and commitment to successful resettlement. Islamophobic and racist sentiment evident in some areas.
Turkey	Classified as a refugee source, destination, and transit country. Currently houses the highest number of refugees in the world (over 3.6 million) with ∼98% in urban areas. Has signed a deal with the EU to prevent onward migration to Europe.	Support offered on the basis of temporary protection regulations. Lack of facilities for rapidly increasing numbers. Many refugees are unregistered so lack access to basic services and protection. Few specialized services for women victims of SGBV.	High profile rape and femicide cases have led to calls for women to be protected from men. Feminist activists have highlighted the need for gender equality. Definitions of SGBV are contested.
Tunisia	In 2019 recorded 1,732 asylum claims. A host to migrants and refugees, mainly from sub-Saharan Africa, fleeing war-torn Libya. A recipient of survivors from the Mediterranean rescue operations requiring immediate medical attention.	Lack of national migration law and policy. No entitlement to public support, education, and work. Refugee claims and shelters coordinated by international NGOs who also promote return migration. Many migrants are unregistered. NGOs lead refugee support.	Most of the migrants transiting through Libya recorded to be survivors of nonpartner sexual violence. A handful of NGOs provides medical and food assistance. Health services available, often for a basic fee. Lack of SGBV definition. Recorded incidents of racism and antimigrant sentiments.

*Note*. SGBV = sexual and gender-based violence; EU = European Union; NGOs = nongovernmental organizations.
